# Acute Thromboembolic Ischemic Stroke From Complex Aortic Arch Plaque

**DOI:** 10.7759/cureus.16977

**Published:** 2021-08-07

**Authors:** Liaquat Ali, Abeer Safan, Sadat Kamran, Naveed Akhtar, Osama Elalamy

**Affiliations:** 1 Neurology, Hamad General Hospital, Doha, QAT; 2 Neurology, Hamad Medical Corporation, Doha, QAT

**Keywords:** atherosclerosis, mechanical thrombectomy (mt), tissue plasminogen activator (tpa), transesophageal echocardiography (tee), low molecular weight heparin (lmwh)

## Abstract

Atherosclerosis is a systemic pathologic process, may involve aorta and is important cause of systemic embolization. The risk of embolism is increased for mobile and complex aortic plaques that are >4 mm thick. The most common manifestations are stroke, transient ischemic attack (TIA) and peripheral embolization. Imaging modalities used include transesophageal echocardiogram (TEE), CT angiography and magnetic resonance angiography (MRA). The mainstays of medical treatment are antiplatelets and statin. The role of anticoagulation is reserved for plaques with thrombotic component.

There were two patients who presented with large acute ischemic stroke with high grade, floating aortic arch thrombus and complex aortic arch plaques. In one of cases, after 10-day follow-up CT aortic angiography showed completely resolved thrombus after being treated with IV tissue plasminogen activator (TPA) followed by low molecular weight heparin (LMWH). The risk of embolism depends on size of aortic plaques and mobility. TEE is modality of choice for thoracic aortic plaques. Aortic plaques >4 mm are independent predictors of recurrent ischemic stroke. There are limited data available for off-label use of intravenous thrombolysis and mechanical thrombectomy (MT) in presence of aortic arch thrombus in acute ischemic strokes. These two case reports help in recognition of aortic arch complex plaques as independent risk factor for recurrent stroke. The right patients may consider about the use of intravenous alteplase and MT performed via trans-brachial access after excluding aortic dissection and aneurysm. In future, multicenter, randomized controlled trials will be required for safety of IV TPA and MT.

## Introduction

Globally, stroke is the second most common etiology of mortality and morbidity [1, 2]. Cryptogenic stroke is considered to be 25 to 40% of ischemic stroke [3]. Atherosclerosis is a pathologic process that causes coronary, cerebral, peripheral artery and aorta disease [4-6]. Aortic atherosclerotic plaques are a manifestation of systemic atherosclerosis [7]. Aortic atherosclerotic plaques are an important cause of systemic embolization [8]. Embolic events in aortic atherosclerosis may occur spontaneously or induced by mechanical interventions during cardiac and vascular surgery [9]. The risk of embolism in patients with aortic atherosclerosis is increased for plaques that are mobile and/or protruding, particularly if >4 mm in thickness.

Thromboembolism from aortic plaques is common and occurs when an atherosclerotic plaque from large or medium arteries becomes unstable, and superimposed thrombi embolize resulting most frequently in stroke or transient ischemic attack (TIA), limb ischemia, renal infarction, intestinal ischemia, and ischemia of other organs. The incidence of recurrent stroke is 11.9% and all vascular event is 26% per year for patients with plaque thickness of >4 mm [8-10]. Complex aortic plaque is defined as thickness >4 mm, ulcerated, or mobility of components of the plaque. The presence of mobile components or ulceration and the absence of calcification in the atheroma are associated with a higher risk of thromboembolism [11, 12]. Complex aortic plaque is seen in 2 to 14% of patients with a history of stroke or peripheral embolization. Twenty-one to 27% of complex thoracic aortic plaque is seen in stroke patients compared with 5 to 9% in controls without stroke in both transesophageal echocardiogram (TEE) and autopsy studies (21 to 27% versus 5 to 9%) [13]. The hypothesis for this retrospective case series was to determine that the complex aortic arch plaque is a risk factor for acute thromboembolic ischemic stroke and safety of IV tissue plasminogen activator (TPA) in these cases.

## Case presentation

Case 1

**Figure 1:**
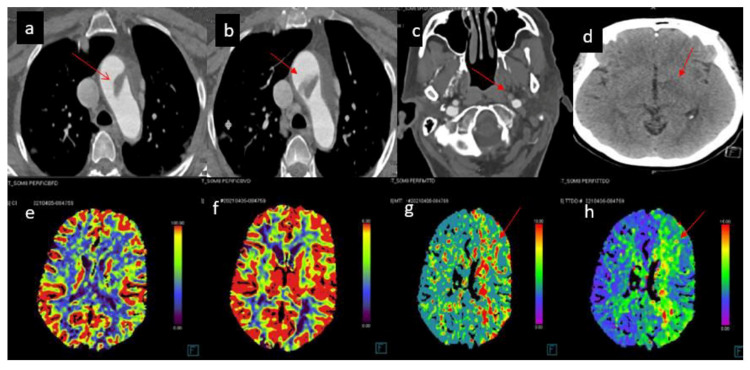
CT head, perfusion and angiography showed a large 2.2-cm floating thrombus within the proximal aortic arch attached to the anterior wall (a, b), and left parietal acute ischemic stroke, left side ICA occlusion and focal severe stenosis at proximal left MCA-M1 segment causing left cerebral hemisphere small infarcted cores, penumbra and other areas of delayed perfusion (c-h). MCA: Middle cerebral artery; ICA: Internal carotid artery.

**Figure 2:**
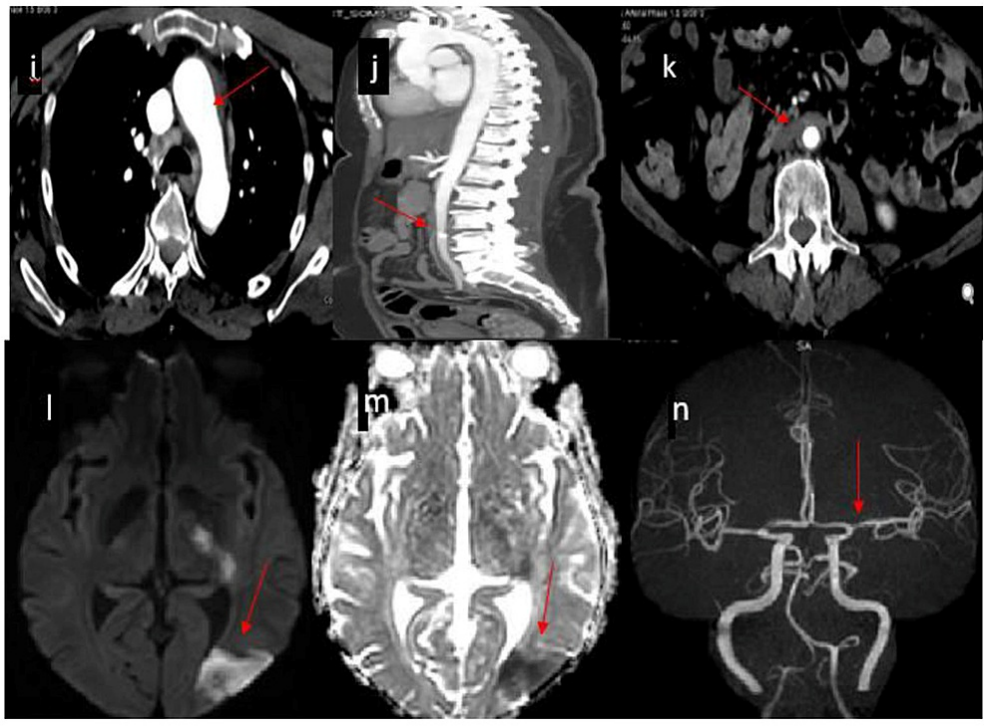
Repeat CT aortic angiography showed diffuse atherosclerotic changes in the aortic arch, descending thoracic and abdominal aorta. These atherosclerotic changes are most severe at the distal part of the descending thoracic aorta which showed circumferential mural thickening and calcification (j, k). No evidence of intraluminal filling defects or floating thrombi (i-n). MRI/MRA head showed left posterior parieto-occipital, left centrum semiovale, left thalamocapsular and left peritrigonal region acute ischemic infarction and left side MCA-M1 segment showed moderate stenosis with mild stenosis proximal basilar artery (l, n). MCA: Middle cerebral artery

A 66-year-old lady with diabetes mellitus (DM) and hypertension (HTN), presented with complaints of slurred speech and right-sided weakness at 0600h morning. She woke up at 0540h and was seen normal on the day of admission. Her Covid-19 polymerase chain reaction (PCR) test turned positive as she had cough. Neurological examination revealed global aphasia, right side lower facial asymmetry, partial left gaze preference, right side strength of 1/5 upper and lower extremity, right homonymous hemianopia, right side neglect with moderate to severe on National Institutes of Health Stroke Scale (NIHSS, score: 14). Initial CT stroke protocol, perfusion and angiography showed left parietal subcortical recent ischemic stroke, left side internal carotid artery (ICA) occlusion and focal severe stenosis at proximal left middle cerebral artery (MCA)-M1 segment causing left cerebral hemisphere small infarcted cores, penumbra and other areas of delayed perfusion. A large 2.2-cm floating thrombus within the proximal aortic arch was attached to the anterior wall. Aortic arch soft and calcified plaques were also seen (Figures 1-2). At 0922h IV thrombolytic bolus alteplase dose was given, continuous infusion of alteplase was withheld because of the doubt about a potential aortic dissection. Intervention radiologist and vascular surgeon were consulted for mechanical thrombectomy or possible surgical intervention, but they recommended that she is not a candidate for intervention due to a chronic intracranial occlusion and continued conservative medical treatment. After 24 hours, repeat non-contrast CT head showed left-sided caudate, and thalamic acute ischemic stroke was noted. MRI/magnetic resonance angiography (MRA) head showed left posterior parieto-occipital, left centrum semiovale, left thalamocapsular and left peritrigonal region ischemic infarction and left side MCA-M1 segment showed moderate stenosis with mild stenosis proximal basilar artery. She was started on low molecular weight heparin (LMWH) therapeutic dosage twice after 24-hour post TPA for five days then switched to oral anticoagulant. Workup for hypercoagulable state was negative except COVID-19 PCR test which was positive (protein C&S activity, anticardiolipin antibodies, factor V Leiden, homocysteine level, activated protein C resistance). Echocardiography showed normal left ventricle (LV) function [Ejection fraction (EF)-65%] and normal valves. Ten days after the stroke, repeat aortic contrast CT angiography showed diffuse atherosclerotic changes in the aortic arch, descending thoracic aorta and abdominal aorta. These atherosclerotic changes were most severe at the distal part of the abdominal aorta which showed circumferential mural thickening and calcifications extending to both common iliac arteries causing mild luminal narrowing. There was no evidence of intraluminal filling defects or floating thrombi, and no evidence of aortic aneurysm or acute aortic dissection. After two weeks of rehabilitation, activity of daily living was dependent on wheelchair and modified Rankin Scale was 4/6 (Moderately severe disability) on discharge.

Case 2

**Figure 3:**
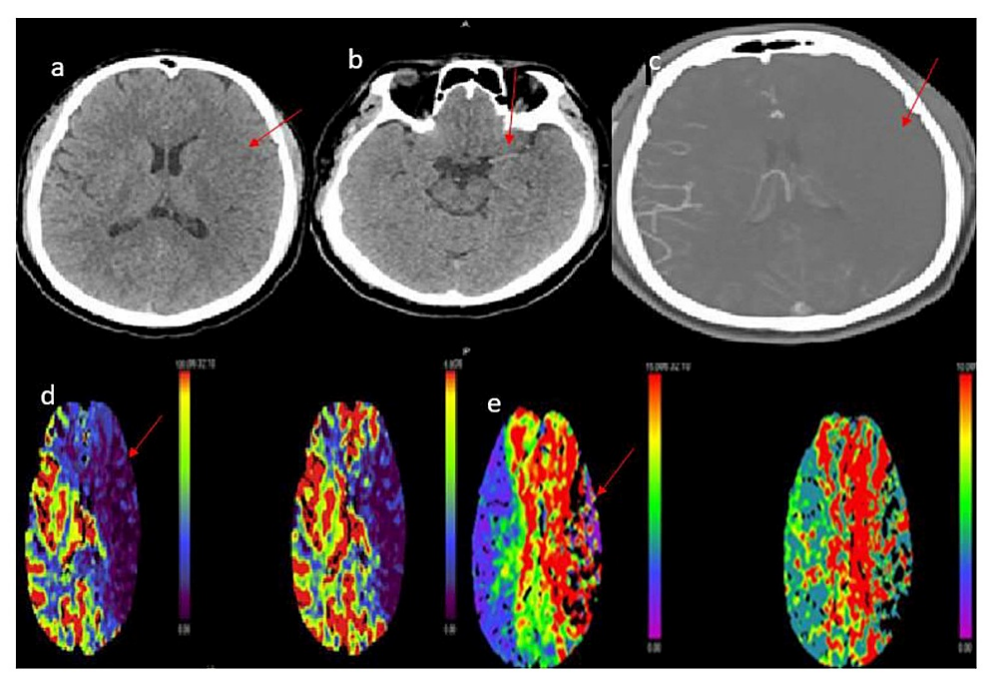
CT head, perfusion and angiography showed left acute infarct of MCA and left capsule-ganglionic region with matched perfusion defect, and right paramedian frontal and parietal lobes showed penumbra (d-e), left MCA dense sign and no collateral vessels visible to the left MCA ischemic site (a-c). MCA: Middle cerebral artery

**Figure 4:**
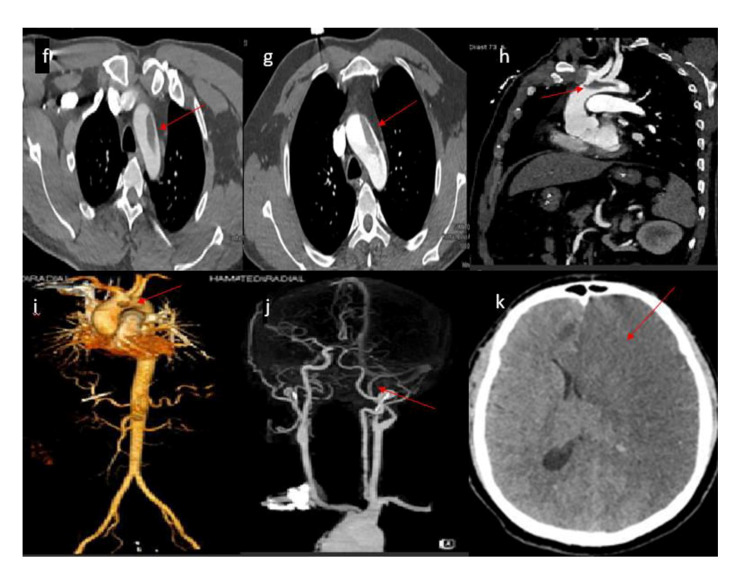
CT angiography showed a large 5 x 1 cm floating thrombus in the arch of the aorta attached to the left anterolateral wall (f-i). CT angiography showed left ICA occlusion petrous and inter-cavernous portion and significant attenuation of the M1 and M2 portion of left MCA (j). CT head showed large left MCA acute ischemic stroke and a small right frontal parafalcine region and 7 mm midline shift to the right side with mass effect (k). MCA: Middle cerebral artery; ICA: Internal carotid artery.

A 39-year-old gentleman with HTN, was brought by emergency medical services (EMS) after he had minor car accident at about 0510h morning. He was noticed to have right side weakness and inability to talk. He reached hospital at 0616h. Covid-19 PCR test was negative. Neurological examination revealed drowsiness, global aphasia, right side lower facial asymmetry, left gaze preference, right side strength of 0/5 upper and lower extremity, right homonymous hemianopia, right side impaired sensations with severe NIHSS (score: 23). Initial CT stroke protocol, perfusion and angiography showed acute infarct involving left frontotemporal parietal lobes and left capsule-ganglionic regions with matched perfusion defect, and right paramedian aspects of frontal and parietal lobes showed mismatched defect (Figures 3-4). CT cerebral angiogram showed left internal carotid artery (ICA) occlusion and significant attenuation of left middle cerebral artery (MCA-M1,M2) with hypoplastic right anterior cerebral artery (ACA-A1). A large 5 x 1 cm thrombus in the arch of the aorta was attached to the left anterolateral wall. No evidence of distal embolus in the visualized thoracic, abdominal or pelvic viscera was noted. The patient had ischemic stroke and was not a candidate for IV thrombolysis and thrombectomy. Workup for hypercoagulable state showed mild positive lupus anticoagulant value of 53.8 seconds (normal 45.3 second), while antineutrophil cytoplasmic antibody (ANCA), anticardiolipin antibodies, factor V Leiden, prothrombin C (factor II), antithrombin activity, protein C & S activity, antibeta-2 glycoprotein were normal. Echocardiography showed normal LV function (EF-52%) and no evidence of thrombus in left ventricle. The patient was admitted in MICU for close monitoring for intracranial pressure (ICP) and neurosurgical intervention. Patient's Glasgow coma scale (GCS) dropped with right anisocoria and he was intubated and ventilated. Repeat CT scan head after 24 hours showed large left MCA full territory and a small right frontal parafalcine acute ischemic stroke. There was a 7-mm midline shift to the right side with mass effect. He underwent decompressive craniectomy. The patient developed diabetic insipidus and acute kidney injury (AKI). He was hemodialyzed for hyperkalemia. On 9th day of admission, he had fixed dilated pupils and absent brainstem reflexes, and he had sudden bradycardia and cardiac arrest and declared dead.

## Discussion

Aortic atherosclerotic complex plaques are important cause of systemic thrombo-embolization that occurs spontaneously or is induced by vascular interventions. The risk of embolism is significantly increased for plaques that are mobile and or protruding, particularly if >4 mm in thickness. Aortic complex plaques of >4 mm are independent predictors of recurrent ischemic strokes.

French Study of Aortic Plaques in Stroke (FAPS) included 331 patients with an initial ischemic stroke who underwent TEE [8]. The incidence of recurrent stroke was 2.8, 3.5, and 11.9% per year for patients with plaque thickness of <1, 1 to 3.9, and >4 mm, respectively. After adjustment of risk factors, aortic plaques >4 mm were independent predictors of recurrent ischemic stroke (relative risk 3.8; 95% CI 1.8-7.8) [10]. Stroke occurs more commonly with complex plaque of the ascending aorta and arch of aorta. In a review of 250 patients admitted with ischemic stroke who were compared with consecutive controls, the odds ratio for stroke was 13.8 for patients with plaques >4 mm in the aortic arch compared to 1.5 with such lesions in the descending aorta [13].

The mainstays of medical treatment for atherosclerotic plaques in the aorta are antiplatelet therapy and statin. The role of anticoagulation is reserved for plaques with a majority of thrombotic component as opposed to complex atheromatous plaques. The Aortic Arch Related Cerebral Hazzard (ARCH) trial was randomized with nondisabling ischemic stroke, TIA, peripheral embolism and 4-mm atherosclerotic plaque in the thoracic aorta on TEE [14]. Subjects were randomly assigned to aspirin plus clopidogrel daily or warfarin [International normalized ratio (INR) 2 to 3]. After a median follow-up of 3.4 years, the primary endpoint (composite of ischemic stroke, MI, peripheral embolism, vascular death, or hemorrhagic stroke) occurred significantly less frequently for patients receiving aspirin plus clopidogrel compared with those receiving warfarin (7.6% versus 11.3%). Major hemorrhage occurred in 2.3% of the aspirin plus clopidogrel and 3.4% in the warfarin arm. These differences were not statistically significant [14].

The stroke prevention in atrial fibrillation III (SPAF-III) trial compared adjusted-dose warfarin (to maintain INR of 2 to 3) to low-dose warfarin (INR of 1.2 to 1.5) plus aspirin for the prevention of stroke in 1044 patients with atrial fibrillation with at least one thromboembolic risk factor [15]. A subset of patients (n = 382) underwent observational TEE; in each treatment group, 35% of patients had complex aortic plaque [16]. At a mean follow-up of 1.1 years, the incidence of stroke in patients with complex aortic plaque was 4% in those treated with adjusted-dose warfarin compared with 16% for those on fixed low-dose warfarin plus aspirin. The risk reduction (75%) was the same as in the entire study population, but the absolute benefit was greater (12% versus 6%) because patients with complex plaque were at higher risk [15]. No data have been reported regarding the role of direct oral anticoagulants or novel oral anticoagulants (NOACs) in the management of aortic atheroma. Results from the SPAF III trial of high-risk patients showing significantly higher event rates for intracranial hemorrhage and ischemic stroke in patients treated with fixed low dose warfarin plus aspirin compared with standard adjusted-dose warfarin. The risk was greater in those with a previous thromboembolic event. Mortality associated with complex aortic plaque may be as high as 20% within three years [17].

In this study, there were two patients with high grade complex aortic arch plaque with floating thrombus that causes thromboembolic large left cerebral hemisphere ischemic stroke. In first case, risks for stroke were DM, HTN and COVID-associated coagulopathy while in the second case, risk factors for stroke were HTN and mildly positive lupus anticoagulant. First case came within 4.5 hours window period and received IV alteplase followed by anticoagulant and after 10 days, follow-up repeat CT aortography showed completely resolved large floating thrombus. There are only limited data available for off-label use of intravenous thrombolysis with alteplase and mechanical thrombectomy performed within window period in presence of aortic arch thrombus. This study will help clinicians in recognizing complex aortic arch plaque as risk factor for thromboembolic and recurrent stroke and consider IV tissue plasminogen activator (TPA) and mechanical thrombectomy (MT) within window period. There are no data on the safety of mechanical thrombectomy in presence of a mobile aortic arch thrombus and treatment decision should be made on a case-by-case basis, taking into account stroke etiology, infarct size, latency and severity of stroke.

## Conclusions

Atherosclerosis is a systemic pathologic process and aortic atherosclerotic plaques are important cause of systemic embolization that occurs spontaneously or induced by vascular intervention. The risk of thromboembolism is significantly increased for complex plaques if they are >4 mm in thickness and mobile. This case series will help clinicians to recognize complex aortic arch plaque as a risk factor for thromboembolic and recurrent ischemic stroke and clinicians may consider use of intravenous alteplase and trans-brachial access mechanical thrombectomy (MT) within window period after excluding aortic dissection and aneurysm. In future, multicentral, randomized controlled trials for safety of IV tissue plasminogen activator (TPA) and mechanical thrombectomy are required.
